# The effects of AUT00206, a novel Kv3.1/3.2 potassium channel modulator, on task-based reward system activation: a test of mechanism in schizophrenia

**DOI:** 10.1007/s00213-022-06216-3

**Published:** 2022-09-12

**Authors:** Stephen J. Kaar, Ilinca Angelescu, Matthew M. Nour, Tiago Reis Marques, Alice Sharman, Anil Sajjala, John Hutchison, Philip McGuire, Charles Large, Oliver D. Howes

**Affiliations:** 1 Institute of Psychiatry, Psychology & Neuroscience – King’s College London, 16 De Crespigny Park, Camberwell, London, SE5 8AB UK; 2 Psychiatric Imaging Group, MRC London Institute of Medical Sciences, Hammersmith Hospital, London, W12 0NN UK; 3 Division of Psychology and Mental Health, Faculty of Biology, Medicine, and Health, University of Manchester, Manchester, M13 9WL UK; 4 Greater Manchester Mental Health NHS Foundation Trust, Manchester, UK; 5 Max Planck University College London Centre for Computational Psychiatry and Ageing Research, London, WC1B 5EH UK; 6 Wellcome Trust Centre for Human Neuroimaging, University College London, London, WC1N 3AR UK; 7 Department of Psychiatry, University of Oxford, Oxford, OX3 7JX UK; 8 Autifony Therapeutics Limited, Stevenage, SG1 2FX UK; 9 South London and Maudsley NHS Foundation Trust, London, UK; 10 Institute of Clinical Sciences (ICS), Faculty of Medicine, Imperial College London, London, W12 0NN UK

**Keywords:** Monetary incentive delay, Parvalbumin, Imaging, Psychosis, Therapy

## Abstract

The pathophysiology of schizophrenia involves abnormal reward processing, thought to be due to disrupted striatal and dopaminergic function. Consistent with this hypothesis, functional magnetic resonance imaging (fMRI) studies using the monetary incentive delay (MID) task report hypoactivation in the striatum during reward anticipation in schizophrenia. Dopamine neuron activity is modulated by striatal GABAergic interneurons. GABAergic interneuron firing rates, in turn, are related to conductances in voltage-gated potassium 3.1 (Kv3.1) and 3.2 (Kv3.2) channels, suggesting that targeting Kv3.1/3.2 could augment striatal function during reward processing. Here, we studied the effect of a novel potassium Kv3.1/3.2 channel modulator, AUT00206, on striatal activation in patients with schizophrenia, using the MID task. Each participant completed the MID during fMRI scanning on two occasions: once at baseline, and again following either 4 weeks of AUT00206 or placebo treatment. We found a significant inverse relationship at baseline between symptom severity and reward anticipation-related neural activation in the right associative striatum (*r* = -0.461, *p* = 0.035). Following treatment with AUT00206, there was a significant increase in reward anticipation-related activation in the left associative striatum (t_(13)_ = 4.23, peak-level *p*(FWE) < 0.05)), but no significant effect in the ventral striatum. This provides preliminary evidence that the Kv3.1/3.2 potassium channel modulator, AUT00206, may address reward-related striatal abnormalities in schizophrenia.

**Supplementary Information:**

The online version contains supplementary material available at 10.1007/s00213-022-06216-3.

## Introduction

Schizophrenia has a lifetime prevalence of approximately 1% (McCutcheon et al. 2020) and affects over 21 million people worldwide (Charlson et al. 2018). Current treatments act predominantly as dopamine-2 receptor antagonists (Kaar et al. 2020), yet these are poorly tolerated or ineffective for many patients (Lally et al. 2016; Demjaha et al. 2017) and have limited benefit for negative symptoms, highlighting the need for new treatment approaches (Howes & Kaar 2018).

Motivational impairment contributes to the negative symptoms seen in schizophrenia (Foussias and Remington 2010; Bègue et al. 2020) and has been linked to abnormalities in the neural processing of rewards and other environmental cues (Gold et al. 2008; Winton-Brown et al. 2014). The monetary incentive delay (MID) task indexes reward processing and, in particular, reward anticipation (Oldham et al. 2018). In the task, participants are presented with cues which differentially predict monetary outcomes. When the task is studied in conjunction with functional neuroimaging, anticipation of reward following cue presentation leads to activation in both the ventral striatum, including the nucleus accumbens, and the dorsal/associative striatum including the putamen and caudate (Oldham et al. 2018; Wilson et al. 2018; Jauhar et al. 2021). Striatal activation is thought to represent a reward prediction signal (Diekhof et al. 2012) that guides value-based decision-making and subsequent updating of cue values following outcomes (Koscik et al. 2020; Filimon et al. 2020).

A large meta-analysis of fMRI studies using the MID task found that patients with schizophrenia show bilateral hypoactivation in the ventral striatum during reward anticipation relative to controls (Radua et al. 2015). Further evidence for striatal involvement in schizophrenia comes from molecular imaging studies showing dysregulated dopamine synthesis and release capacity in schizophrenia (Brugger et al. 2020), which is most marked in a part of the dorsal striatum termed the associative striatum because it receives projections from frontal and other associative cortical regions (McCutcheon et al. 2018, 2021). Hypoactivation of the dorsal striatum, in particular the associative striatum, has also been found during the MID fMRI task in patients with schizophrenia (Mucci et al. 2015; Li et al. 2018). Moreover, lower activation in associative striatal regions has been associated with greater symptom severity, particularly the severity of avolition (Mucci et al. 2015), and poorer subsequent response to antipsychotic treatment (Nielsen et al. 2018).

Activity of mesostriatal dopamine neurons is central to reward processing (Schultz et al. 1997) and the firing of mesostriatal dopamine neurons is regulated by striatal GABAergic output neurons (Groenewegen 2003), predominantly in the form of medium spiny neurons (Kawaguchi et al. 1995). In addition, striatal fast-spiking GABA interneurons (Kawaguchi et al. 1995), of which those containing parvalbumin are the best characterised (Hu et al. 2014), receive input via cortico-striatal afferents (Bennett and Bolam 1994) and synapse on medium spiny neurons, to regulate spike timing, and thus striatal output (Tepper et al. 2008; Lee et al. 2017; Gritton et al. 2019). Modulation of the excitability of these GABAergic circuits thus represents a potential therapeutic target for treating reward processing deficits and negative symptoms in schizophrenia.

Kv3.1 and Kv3.2 potassium channels are highly expressed on fast-spiking GABA interneurons including those that express parvalbumin in the striatum (Chow et al. 1999; Rudy and McBain 2001), and Kv3 knockout mice show locomotor hyperactivity, a behavioural phenotype associated with preclinical models of schizophrenia (Espinosa et al. 2004; Kokkinou et al. 2021). In view of this, Kv3.1 and Kv3.2 channels have been proposed as drug targets for modulating GABA neuron activity in schizophrenia (Gargus et al. 1998; Volk & Lewis 2005). Modulation of Kv3.1 and Kv3.2 channels leads to increased firing frequency of fast-spiking GABAergic interneurons and improves gamma oscillation regularity, which is thought to be a marker of the cortical excitation-inhibition balance that is disrupted in schizophrenia (Boddum et al. 2017; Andrade-Talavera et al. 2020). AUT00206 is a modulator of Kv3.1/3.2 channels that has been shown to both enhance whole-cell currents and the power of fast network oscillations (Large et al. 2016). In healthy subjects, it reduced BOLD signal changes in cortical and sub-cortical regions of the brain following ketamine; a psychomimetic agent that induces schizophreniform symptoms (Deakin et al. 2019; Beck et al. 2020), and, in rodents, it reversed the cognitive and behavioural effects of a phencyclidine (PCP) model of schizophrenia (Leger et al. 2014). A compound from the same series, AUT1, also modulates Kv3.1/3.2 channels and has been shown to block the effects of amphetamine induced hyperactivity and to increase inhibition of dopamine neuron firing in the midbrain, suggesting that modulating Kv3.1/3.2 channels could counter overactivity of mesostriatal dopamine neurons (Parekh et al. 2018). These findings, in both preclinical and human models relevant to the pathophysiology of schizophrenia, suggest that AUT00206 could improve aberrant reward processing in the disorder.

To test this, we used fMRI to determine whether striatal activation during reward anticipation is modulated by AUT00206 in patients with schizophrenia. We hypothesised that AUT00206 would increase striatal activation during the reward anticipation phase of the monetary incentive delay (MID) task (Knutson et al. 2000). Our primary regions of interest were the ventral striatum, given the meta-analytic evidence of hypoactivation during this task in schizophrenia as discussed above, and the associative striatum, as the major dopaminergic dysfunction in schizophrenia is localised in this region (McCutcheon et al. 2018) and the evidence discussed above that hypoactivation in this region is associated with symptoms in schizophrenia.

## Materials and methods

We conducted a study using fMRI imaging during a reward anticipation task to provide an initial test of mechanism and address our hypothesis as part of a phase 1b study of the safety and tolerability of AUT00206 in schizophrenia (ClinicalTrials.gov Identifier: NCT03164876). This paper reports on the reward anticipation data only. The primary trial results of the safety and tolerability evaluations are reported elsewhere (ClinicalTrials.gov Identifier: NCT03164876). A placebo group was included for safety monitoring, not for a formal comparison of imaging biomarkers with placebo. However, we also report the results of the imaging in the placebo group for qualitative comparison. The study protocol was approved by the NHS research ethics committee (London Central Research Ethics Committee—17/LO/0066) and appropriate authorities for all sites involved. The study was performed in accordance with the principles stated in the Declaration of Helsinki and Good Clinical Practice guidelines, as applicable at the time. All patients provided written informed consent.

### Participants and procedures

Patients were recruited to participate in the study from the South London and Maudsley NHS Foundation Trust and the Central and North West London NHS Foundation Trust, London, between April 2017 and April 2019 as part of a first in patient study exploring the safety and tolerability of (ClinicalTrials.gov Identifier: NCT03164876)); 24 patients with schizophrenia were randomised in a 2:1 ratio to receive repeated doses of AUT00206 (16 subjects) or matching placebo (PBO) (8 subjects).

Subjects who were randomised to active treatment received a loading dose of 2000 mg AUT00206 on Day 1, followed by repeated twice daily-oral doses of 800 mg AUT00206 on days 2 – 27 and a single oral dose of 800 mg AUT00206 on Day 28. The initial loading dose was chosen to ensure blood levels of AUT00206 were within a target therapeutic range within the first 24 h, based on preclinical data (data on file, Autifony Therapeutics Ltd, Stevenage, UK). AUT00206 and antipsychotic levels were conducted throughout the study to monitor concordance. Subjects underwent functional magnetic resonance imaging (fMRI) on a 3 T (Siemens Verio) MRI scanner at baseline and during treatment (between Day 14 and Day 28).

There was no difference between PBO and AUT groups in the mean duration separating baseline and start of dosing (AUT = 7.93 [SD 6.60], PBO = 8.14 [SD 9.82] days, *t *= -0.06, *p* = 0.95, two sample t-test), or in the mean number of dosing days at time of follow-up scan (AUT = 17.86 [SD 3.9], PBO = 16.43 [SD 2.63] days, *t* = 0.87, *p* = 0.39, two sample t-test).

The inclusion criteria were: male (due to a lack of safety data in females), outpatients, 18–50 years of age who met criteria for schizophrenia (confirmed using the Structured Clinical Interview for DSM-5 Disorders, Clinician Version (SCID-5-CV) (First 2015)), no more than 5 years to have passed since first diagnosis; one positive symptom item score > 3 or 2 or more positive symptoms = 3, and one negative symptom item score > 3 or 2 or more negative symptoms = 3 on the positive and negative syndrome scale (PANSS) (Kay et al. 1987); on a stable dose of 1 or 2 antipsychotic drugs (excluding clozapine) for at least 1 month before screening, and able to give fully informed written consent. No clinically relevant abnormalities in clinical examination or electrocardiography (ECG) findings were allowed.

Exclusion criteria were: severely underweight or morbidly obese people, presence of an acute or chronic illness other than mild, well controlled illnesses, homicidal ideation or intent, suicidal ideation with some intent to act in the last 6 months based on the Columbia-Suicide Severity Rating Scale (C-SSRS) (Posner et al. 2011), moderate or severe depressive or anxiety symptoms as indicated by a score of ≥ 11 on the Hospital Anxiety and Depression Scale (HADS) (Zigmond and Snaith 1983), presence or history of severe drug reaction, alcohol or drug dependence in the last 12 months before admission, or presence of a contradiction to an MRI scan. Concomitant psychotropic medications were permitted unless contraindicated due to their action on the cytochrome p450 (CYP) system.

### Monetary incentive delay (MID) task

**Fig. 1:**
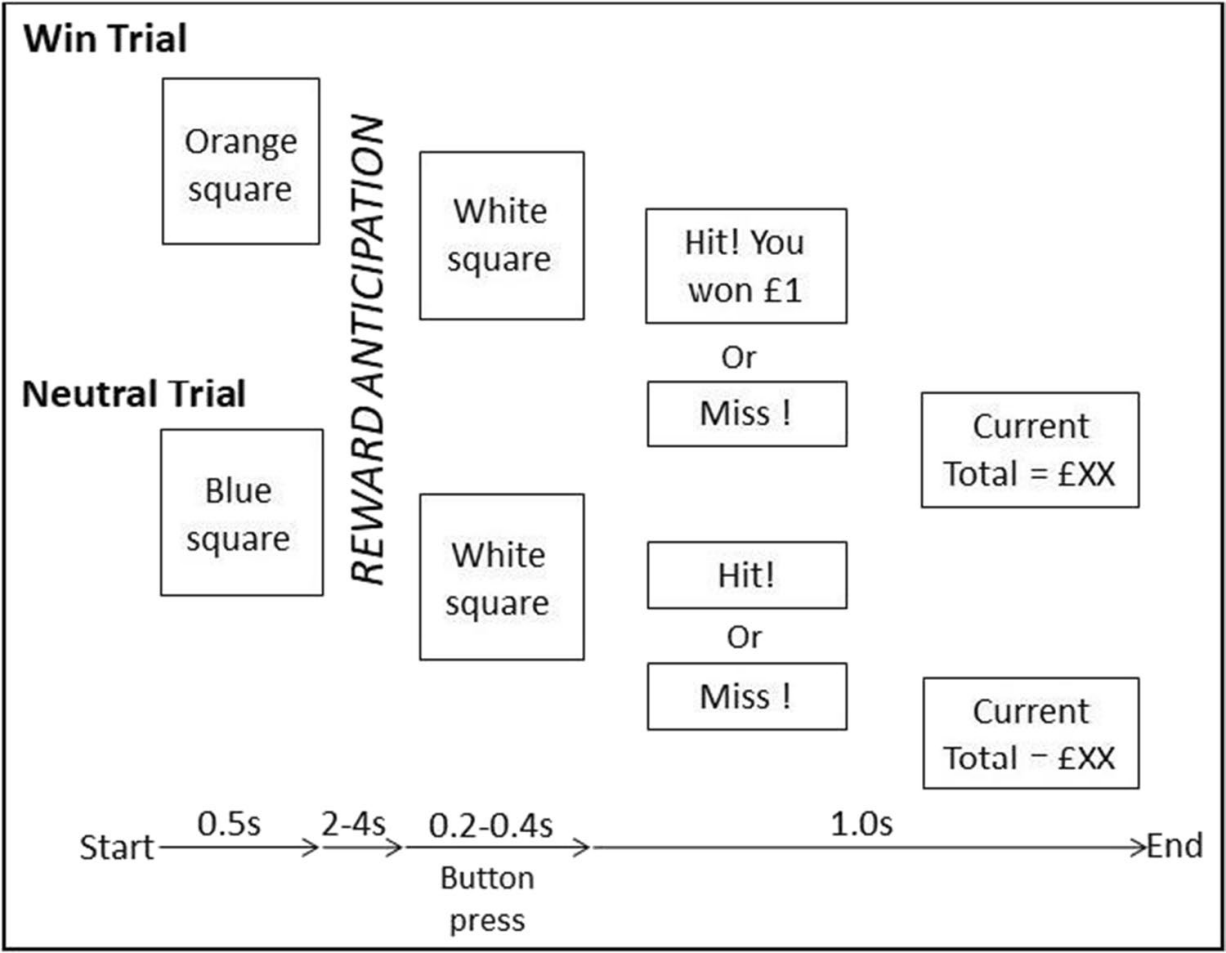
MID task outline showing the two trial types included in the task: win trials (top) and neutral trials (bottom)

All participants completed the MID task (Knutson et al. 2000) on both scanning sessions. The task included two trial types (win or neutral). Each trial began with a cue stimulus (an orange square before a win trial and a blue square before a neutral trial) that lasted 0.5 s. This cue was followed by a variable reward anticipation period (2, 3 or 4 s time interval occurring randomly), after which a target stimulus (a white square) appeared. Participants were instructed to respond to the appearance of the target stimulus as quickly as possible using a button box in the scanner. The target stimulus presentation duration varied with each trial (further details below). Following the target stimulus, feedback on the outcome was presented. During a win trial the participant won £1 if their response to the target was within the allotted response time window. If this occurred, then the message “Hit! You won £1” was shown on the subsequent feedback screen in green text for 1000 ms. On neutral trials, it was not possible to win money; however, if the participant’s response occurred during target presentation, the subsequent feedback screen displayed “Hit!” in green text for 1000 ms. If the participant failed to respond to the target stimulus in time, then the feedback screen showed “Miss” in red text for 1000 ms. A second feedback screen always appeared after the first feedback was given, to inform the participant of the running total of winnings, e.g. “Current Total = £XX”. See Fig. 1 .

The target stimulus duration was dynamically adjusted to ensure all participants experienced a similar level of difficulty and reward rate. Specifically, missed responses during target presentation led to an additional 16.66 ms of target duration on the subsequent trial. Correct responses led to the subtraction of 16.66 ms of target duration on the subsequent trial. The target stimulus duration began at 300 ms and could increase up to 400 ms and decrease down to 200 ms over the course of the task. The feedback stimulus duration also increased or decreased in proportion to the target stimulus duration change, such that the total duration of time including target stimulus and feedback was maintained at 1300 ms. The inter-trial interval which lasted from feedback to next cue stimulus onset consisted of a fixation point presentation and the duration was randomly manner varied using a Poisson distribution between 2.2 and 10.2 s in one second intervals (Hagberg et al. 2001). The task lasted for 12 min (with a 10 s buffer period at the end) in total, resulting in 608 scanning volumes. In total, there were 24 win trials and 48 neutral trials (mirroring the win–neutral asymmetry in earlier versions of the task (Knutson et al. 2000)).

### fMRI acquisition

The T1-weighted anatomical scan used a Magnetisation Prepared Rapid Gradient Echo (MPRAGE) sequence using parameters based on the Alzheimer's Disease Research Network sequence (ADNI-GO; 160 slices × 240 × 256, TR = 2300 ms, TE = 2.98 ms, flip angle 9°, 1 mm isotropic voxels, band- width = 240 Hz/pixel, parallel imaging factor = 2, inversion time = 900 ms) (Jack et al. 2008). The MID sequence was based on the multiband echo-planar imaging (Siemens WIP v012b) provided by the University of Minnesota (Setsompop et al. 2012) and used a multiband acceleration factor of 2, for simultaneous acquisition of 2 slices. Other characteristics of the sequences were as follows: TR = 1200 ms, TE = 30 ms, 42 axial slices, 3 mm isotropic voxels, FOV = 192 mm, bandwidth = 1906 Hz/pixel, parallel imaging factor = 2, flip angle = 62°, echo spacing = 0.61 ms.

### Behavioural statistical analysis

To quantify motivational salience during task performance, we quantified (1) the difference between the percentage of hits during win trials compared to neutral trials and (2) the difference in mean reaction time (RT) between win and neutral trials. A one-sample t-test was performed to confirm that subjects showed a main behavioural effect of cue value at baseline and a paired t-test was used to compare the effects of treatment on behavioural measures in the AUT00206 and PBO groups.

### fMRI imaging and statistical analysis

#### Pre-processing

We employed a standard fMRI spatial pre-processing pipeline implemented in the SPM toolbox (version 12–6906) for MATLAB (version 9.2.0). For each scanning session, this included motion correction by realignment of functional scans to the first volume, co-registration of functional to the T1 structural image, and normalisation of the images to the standard template provided by the Montreal Neurological Institute (MNI template) using the DARTEL routine (Ashburner 2007). The quality of the normalisation was manually checked for each subject. Normalised functional images were spatially smoothed using a 6 mm full width at half maximum Gaussian kernel. Functional images were resampled to 1.5 mm isotropic voxels.

#### Analysis of reward anticipation fMRI activation

At the first (single subject) level, we implemented a standard mass-univariate general linear model (GLM) analysis approach for fMRI analysis, as implemented in SPM12 (http://www.fil.ion.ucl.ac.uk/spm) (Penny et al. 2007). This GLM included stick regressors for cue onset and target onset. Cue onset was parametrically modulated according to whether the cue was predictive of a win (1) or neutral (0) outcome (equivalent to a contrast of reward predicting vs. neutral cue). Regressors were convolved with the canonical haemodynamic response function. The first level GLM also included nuisance regressors for six head motion realignment parameters to minimise the influence of head movement on individual participant BOLD activation estimates. Of note, a formal comparison of mean and maximum framewise displacement measures between groups and sessions (i.e. group*session ANOVA) revealed no significant main effects of group, session, or group*session interaction on head motion variables (all *p* > 0.05). We additionally included temporal derivatives (first-order differences), applied an autoregressive model (to account for serial correlations in the fMRI time series), and used a 128 s cut-off high-pass filter. The analysis was performed on each separate scanning session for each subject. Within each scanning session, our contrast of interest was the voxel-wise beta estimate associated with the parametric modulator at cue onset (${\beta }_{rew})$, which indexes the degree to which a voxel’s BOLD response is higher for reward predicting vs neutral cues (i.e. ‘reward anticipation’).

We first tested if the task resulted in significant ‘reward anticipation’ related activation (i.e. ${\beta }_{rew}$ effect) in the whole sample (*n* = 21) at baseline using a one-sample t-test at the second (group) level. To test the relationship between reward anticipation activation and symptoms, we correlated the ${\beta }_{rew}$ effect with PANSS total symptom scores at baseline, in the whole sample (*n* = 21). Here, we extracted the mean ROI voxel parameter estimates for reward anticipation (${\beta }_{rew})$ from pre-defined anatomical regions of interest (ROI, see below) using the MarsBaR toolbox (http://marsbar.sourceforge.net/) in SPM. We then used Pearson’s product moment correlation to test the correlation between ${\beta }_{rew}$ and PANSS symptoms in each ROI.

Our primary hypothesis was that AUT00206 would increase reward anticipation-related ventral striatal activation (i.e. ${\beta }_{rew})$ from session 1 (baseline) to session 2 (on drug). To test this, for each subject we calculated an ‘activation change’ statistical image, ${\Delta \beta }_{rew}= {\beta }_{rew}\left(scan2\right)- {\beta }_{rew}\left(scan1\right),$ indexing the change in BOLD activation for reward anticipation from baseline to treatment scan at each voxel, with a positive value indicating a drug-related increase. In the second level (group) analysis, we then conducted a one-sample t-test of the activation change images in the AUT00206 group with the ventral striatum as the a priori region of interest (ROI), based on meta-analytic evidence that the ventral striatum is hypoactive in patients compared with controls during reward anticipation tasks (Radua et al. 2015). We defined left and right sided ventral striatal (VS) ROIs as 8-mm radius spheres centred on the ventral striatal coordinates of the peak activation reported in the meta-analysis (Knutson and Greer 2008) and converted them to MNI space using MNI2TAL software (BioImage Suite Web 2021). We used this meta-analysis to define the ROI because it is widely used in the field of schizophrenia research (Yip et al. 2015; Kirschner et al. 2018; Hawkins et al. 2021), which aids the comparison of our findings to previous work. We also conducted an analysis using left and right sided associative striatal ROIs, a region involved in reward anticipation (Oldham et al. 2018) and schizophrenia dopaminergic pathophysiology (McCutcheon et al. 2018), using the MNI coordinates for the associative striatum (AS) as used in the studies in schizophrenia (Martinez et al. 2003; Howes et al. 2009, 2013; Kegeles et al. 2010; Mizrahi et al. 2012; Sorg et al. 2013) to create 8 mm radius sphere ROIs. The ROIs were used to restrict the analysis to the regions of interest (MNI coordinates: Left VS -12, 13, -6, Right VS 11, 11, -4, AS ± 24, 12, 0.) A one-sample t-test was used to determine if there were significant activation change images in the AUT00206 group. The same approach was repeated for the placebo group.

This study was not powered for between group analyses; however, in light of our findings in the AUT00206 group, we undertook an exploratory between treatment group analysis using mean voxel parameter estimates for the ROIs in the ventral and associative striatum at baseline and follow-up, to test whether change in activation during reward anticipation was significantly different between the AUT00206 and PBO treatment groups. We used an independent samples t-test on the change in activation between groups. Further ROI details are found in Table 1 of the supplementary information, and the extracted mean ROI voxel parameter estimates for each group (AUT00206 or PBO) in each ROI at baseline and on-treatment are reported in supplementary table 3.

When reporting whole-brain activation contrasts, we use a cluster forming threshold set to *p* < 0.001 (uncorrected) and we report activations that surpass a whole-brain cluster-corrected significance threshold *p*(FWE)_cluster_ < 0.05 at whole-brain cluster level (Eklund et al. 2016). When reporting activations within a priori anatomical regions (ROIs), we report activations that surpass small-volume-corrected peak level significance thresholds *p*(FWE)_peak_ < 0.05.

## Results

### Clinical characteristics

Of the 24 patients with schizophrenia who were randomised to receive AUT00206 (*n* = 16) or placebo (*n* = 8), three were excluded due to claustrophobia leading them to withdraw from participating in subsequent scans. This left 21 patients with complete MID datasets (*n *= 14 patients who received AUT00206 and *n* = 7 who received placebo). All patients received their assigned treatment from Day 1 to Day 28, except one patient in the AUT00206 group, who received his treatment from Day 1 to Day 21 only and was scanned at this point before withdrawing from the study and stopping treatment. On PK sampling, the median Cmax for AUT00206 was 3745 ng/mL and the mean Ctrough (Cpre-dose) values were > 2300 ng/mL on Days 4–6, ~ 1800 ng/mL on Day 14 and ~ 2200 ng/mL on Days 21 and 28. Target concentrations, based on preclinical models and the ketamine challenge study in HVs, were between 1500 and 4000 ng/mL. Table 1 shows the baseline demographic and clinical characteristics of the patients (*n* = 21) who completed the MID fMRI task at baseline and on treatment.

**Table 1:** Baseline demographic and clinical characteristics of the MID fMRI population. Significance of categorical variable group difference was measured using the Chi -square test (1) and for numerical variable group difference significance was assessed using an independent sample *t*-test (2)

	AUT00206	PBO	
	*n* = 14	*n* = 7	
Male n(%)	14 (100)	7 (100)	
Ethnicity: black n(%)	10 (71)	5 (71)	
Ethnicity: white n(%)	2 (14)	2(29)	
Ethnicity: Asian n(%)	1 (7)	0	
Ethnicity: other n(%)	1 (7)	0	*p* = 0.7^1^
Age (mean(sd))	28.4 (± 6.23)	29.1 (± 5.49)	*p* = 0.9^2^
Chlorpromazine (CPZ) equivalent dose/mg per day (mean(sd))	252.6 (± 148.6)	181.6 (± 40.0)	*p* = 0.2^2^
1st generation antipsychotic n(%)	2 (14.3)	0 (0)	
2nd generation antipsychotic n(%)	7 (50.0)	5 (71.4)	
3rd generation antipsychotic n(%)	4 (28.6)	2 (28.6)	
Combination antipsychotics n(%)	1 (7.1)	0 (0)	*p* = 0.6^1^
Baseline PANSS total mean(sd)	79.6 (± 11.7)	76.4 (± 8.1)	*p* = 0.5^2^
Baseline PANSS positive mean(sd)	19.7 (± 4.6)	18.9 (± 1.9)	*p* = 0.7^2^
Baseline PANSS negative mean(sd)	20.4 (± 4.2)	20.4 (± 4.3)	*p* = 0.9^2^
CGI mean(sd)	3.5 (± 0.65)	3.6 (± 0.53)	*p* = 0.8^2^

### Behavioural

In the whole group, percentage hit rate was higher for win trials than neutral trials at both baseline (mean %hit in win =  60.1, SD 1.7, neutral = 43.7, SD 10.4, *t*_(20)_ = 4.1, *p* = 0.001, one-sample t-test) and follow-up (mean %hit in win = 60.1, SD 14.6, neutral = 40.7, SD = 14.4, t_(20)_ = 6.33, *p* = 0.001, one-sample t-test). Reaction time (RT) was shorter during win trials than neutral trials at both baseline (mean RT win = 233.82 ms, SD 40.49 ms, neutral = 240.46 ms, SD 47.43 ms, *t*_(20)_ = -2.71, *p* = 0.013, one-sample t-test) and follow-up (mean RT win = 229.30 ms, SD 49.69 ms, neutral = 235.07 ms, SD 51.14 ms, *t*_(20)_ = -2.33, *p* = 0.03, one-sample t-test). There was no significant difference between baseline and follow-up performance in the AUT00206 group (*n* = 14, *p* > 0.48 for all measures) or the placebo group (*n* = 7, *p* > 0.8 for all measures). These results confirm a significant reward-related motivational salience effect during both scanning sessions in our sample.

### fMRI

#### Baseline reward anticipation (win vs neutral)

We first confirmed in the whole sample (*n* = 21) at baseline (scan 1) that the MID task was associated with reward anticipation-related BOLD activation (${\beta }_{rew}$) in the striatum. We found significant peak level activation at *p*(FWE) < 0.05 following small volume correction in the bilateral ventral and associative striatum ROIs (*t*_(20)_ = 3.94, *p*(FWE)_peak_ = 0.027 [MNI 12,4, -2] and *t*_(20)_ = 3.57, *p*(FWE)_peak_ = 0.041 [MNI -28, 16,2], respectively). We additionally examined reward anticipation-related BOLD activation (${\beta }_{rew}$) at baseline at whole-brain cluster-corrected *p*(FWE)_cluster_ < 0.05, finding widespread activation also encompassing striatal regions (see supplementary table 2 and Fig. 3a).

**Fig. 2:**
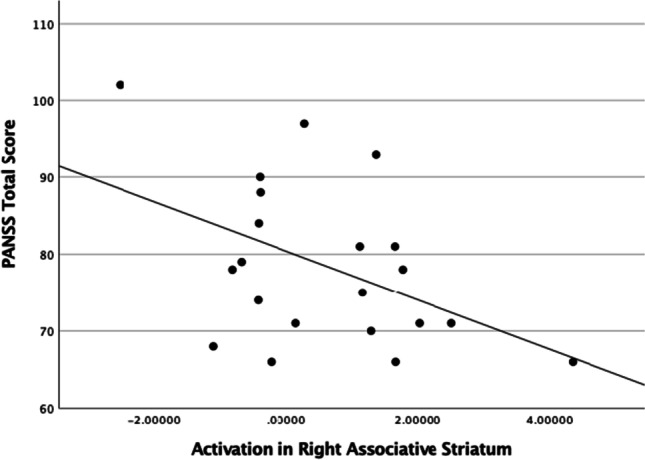
Activation during reward anticipation (${\beta }_{rew}=$ win > neutral contrast) in the right associative striatum (ROI MNI coordinates: 24, 12, 1) at baseline (*n *= 21), showed a significant negative correlation (*n* = 21 *r *= -0.461 *p* = 0.035) with baseline PANSS total score

In addition, across all participants, there was a significant negative correlation between PANSS total score and reward anticipation-related activation (${\beta }_{rew}$) in the right associative striatum at baseline scan (*n* = 21, *r* = -0.461, *p* = 0.035, see Fig. 2) and a trend correlation in the left associative striatum (*n* = 21, *r* = -0.411, *p* = 0.064), but no significant relationship between positive or negative symptoms (PANSS positive or negative sub-scale scores) and right or left ventral striatal activation (${\beta }_{rew}$) at baseline. These findings did not survive Bonferroni correction for multiple comparisons.

#### AUT00206-related increase in reward-related striatal activation

**Fig. 3:**
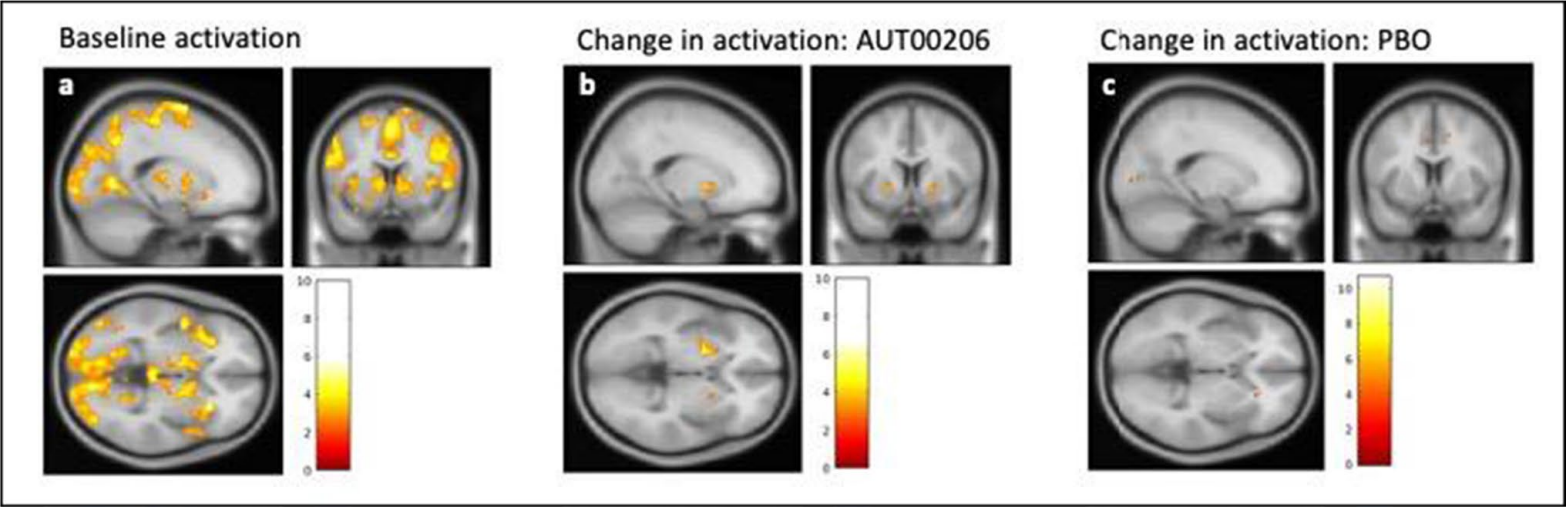
Activation during reward anticipation (${\beta }_{rew}=$ win > neutral contrast), and change in activation following treatment (${\Delta \beta }_{rew}$); 3a: Whole brain analysis in the whole sample at baseline (*n* = 21), showing bilateral striatal activation for reward anticipation at cue onset (${\beta }_{rew}$ regressor); 3b: AUT00206 group (*n* = 14) (${\beta }_{rew}$(follow-up) – ${\beta }_{rew}$(baseline) activation whole brain change image) showing increased activation in the striatum following treatment with AUT00206 (significant at P(FWE) < 0.05 voxel-level following small volume correction, see main text); and 3c: PBO group (*n* = 7) (${\beta }_{rew}$(follow-up) – ${\beta }_{rew}$(baseline) activation whole brain change image). There was no significant change in striatal activation following treatment with placebo at *p*(FWE) < 0.05 significance thresholds (see main text). All images threshold set at *p* = 0.01 uncorrected with cluster defining size of 30 voxels, for illustration purposes only (see main text for family-wise error corrected statistical results). Section orientated to the left dorsal striatum (MNI coordinate -18,8,-2). The colour bar shows the *t* statistic

We next compared reward-related BOLD activation between baseline and on treatment scans in the AUT00206 group (i.e. the activation change measure, ${\Delta \beta }_{rew}$= ${\beta }_{rew}\left(scan2\right)- {\beta }_{rew}\left(scan1\right)$). There was no significant difference in ${\Delta \beta }_{rew}$ in the striatum, either in a whole brain analysis (no clusters surpassing whole-brain cluster-corrected significance threshold of *p*(FWE)_cluster_ < 0.05) or in small volume correction analyses using left or right ventral striatum ROIs (no voxels surpassing *p*(FWE)_peak_ < 0.05). However, when conducting a small-volume correction analysis in the associative striatum, we found a significant increase in activation in the left associative striatum following AUT00206 treatment (i.e. positive ${\Delta \beta }_{rew}$) (t_(13)_ = 4.23, *p*(FWE)_peak_ = 0.04, [MNI -20, 6, -2]) that was not present in the right associative striatum *(p*(FWE)_peak_ values > 0.05). No extra-striatal brain regions showed significant changes with AUT00206 treatment in the whole brain analyses. There was no significant change in activation in the placebo group in either left or right associative or ventral striatum (all *p*(FWE) values > 0.05), and this was also the case on the whole brain analyses. See Fig. 3b and c for an illustration of the activation change following treatment in the AUT00206 and PBO groups.

Finally, we conducted an exploratory between-group analysis to examine whether ${\Delta \beta }_{rew}$ was significantly different under AUT00206 vs placebo in striatal ROIs (i.e. whether the change in ${\beta }_{rew}$ from scan 1 to scan 2 was significantly different between groups). For each participant, we extracted mean ${\Delta \beta }_{rew}$ values for voxels within pre-specified ROIs (Associative Striatum (AS) and Ventral Striatum (VS)) and compared treatment and placebo groups using two-sample *t*-tests. We found a trend group difference in ${\Delta \beta }_{rew}$ in the right associative striatum (*t*_(19)_ = 1.816, *p* = 0.085). In this ROI, the AUT00206 group showed an increase in activation following treatment (*n* = 14, mean change = 0.91, SEM = 0.387) which was not seen the PBO group (*n* = 7, mean change = -0.19, SEM = 0.357). We found no significant group differences in the other 3 ROIs (left AS and right/left VS). In a supplementary analysis of covariance (ANCOVA), we found no evidence that the number of dosing days completed at the time of the follow-up scan predicts variance in right AS activation (*p*-value of covariate = 0.06). See supplementary table 3 for the extracted mean beta values for each group in each ROI at baseline and following treatment.

## Discussion

We found increased activation during reward anticipation in the left associative striatum in patients with schizophrenia receiving treatment with AUT00206. These findings extend previous preclinical and healthy volunteer evidence that AUT00206 engages striatal circuits relevant to the pathophysiology of schizophrenia (Deakin et al. 2019), to show this for the first time in patients with schizophrenia. Hypoactivation in the dorsal striatum, which contains the associative striatum, during reward anticipation has been found in people with chronic schizophrenia and to correlate with symptoms (Mucci et al. 2015; Stepien et al. 2018). Our findings add to these data by showing a correlation between greater hypoactivation in the right associative striatum during reward anticipation and higher total PANSS symptoms in a sample of patients within five years of illness onset. This adds to evidence that altered dopamine function in the associative striatum is associated with symptoms early in the course of schizophrenia (Jauhar et al. 2019). Our results also extend prior findings by showing that treatment with a drug with no appreciable dopamine D2 receptor blockade, AUT00206, is associated with increases in striatal activation in the left associative striatum. In contrast, we did not find a significant effect of AUT00206 on ventral striatal activation. Longitudinal studies have found that treatment with antipsychotics can increase the attenuated activation in patients with schizophenia in the ventral striatum during reward anticipation fMRI tasks (Nielsen et al. 2012; Wulff et al. 2019). As patients in our study were receiving antipsychotic treatment, this may explain why we did not find a change in the ventral striatum in our sample, who were all taking antipsychotic medication, and highlights the need to test AUT00206 in unmedicated patients with schizophrenia to determine if this explains the absence of an effect of AUT00206 in this region.

### Strengths and limitations

This is the first study in patients with schizophrenia to explore the effects of a Kv3.1/3.2 potassium channel modulator on neural activation during reward anticipation. The results demonstrate good subject engagment with the task and activation of the striatum at baseline. A strength is that all patients were diagnosed with schizophrenia using the SCID and had no alcohol or drug dependence. A limitation is the modest sample size. The sample was limited because it was the first study of the drug in schizophrenia, and the study was an add-on to a study primarily aimed at safety assessment. As such, the study was not powered for AUT00206 vs placebo comparisons. Thus, although we did not observe striatal changes in the placebo group and found a trend for a signficant difference between placebo and AUT00206 effects, future studies with larger sample sizes are required to conclude that AUT00206 alters striatal reward processing to a greater degree than placebo, with confidence. Moreover, as this was an initial exploratory study, we did not correct the statistical threshold for the number of primary regions of interest. A further consideration is that of BOLD signal drop out, which degrades signal-to-noise ratio. We found no significant AUT00206 effects in the ventral striatum, which could be a consequence of low statistical power, stemming in part from low within-participant signal-to-noise ratio in this region. Additionally, we used a well cited meta-analysis (Knutson and Greer 2008) to define our ventral striatal ROI coordinates, which facilitates comparison with previous studies in schizophrenia. However, we recognise that these coordinates differ from those reported in a recent meta-analysis in schizophrenia (Jauhar et al. 2021). As such, the results should be considered preliminary. Patients were also already medicated with antipsychotics, which, given they may modulate striatal activity, could have reduced the capacity to detect effects of adjunctive AUT00206. These issues highlight the importance of carrying out a further study in a larger sample, ideally in patients who are medication-naïve or medication-free.

#### Mode of action of AUT00206

AUT00206 is a positive modulator of Kv3.1 and Kv3.2 channels (Rudy and McBain 2001). Kv3.1 channels are expressed by parvalbumin (PV)-positive GABA interneurons that regulate striatal activation and striatal output neurons (Lenz et al. 1994; Weiser et al. 1994, 1995; Chow et al. 1999; Jinno and Kosaka 2004; Yanagi et al. 2014). Parvalbumin interneurons in the striatum synapse onto medium spiny neurons (Lee et al. 2017). Medium spiny neurons are the major cell type in the striatum, and also receive inputs from dopaminergic projections from the midbrain (Tepper et al. 2018). Fast-spiking parvalbumin interneurons exhibit a ﻿pre-reward ramping increment in firing rate (Lansink et al. 2010) and are thought to exert inhibitory control over medium spiny projection neurons in the striatum (Koós and Tepper 1999; Assous et al. 2019). Lower levels of markers for PV interneurons are found in post-mortem brain samples from patients with schizophrenia, although this has not beenspecifically investigated in the striatum (Curley & Lewis 2012; Kaar et al. 2019). AUT00206 enhances the activity of PV interneurons and rescues a range of behavioural deficits in rats previously treated with sub-chronic phencyclidine (Leger et al. 2014). Thus, one plausible explanation for our findings is that AUT00206 is acting to increase PV interneuron activity in striatum to fine-tune the striatal response during reward anticipation.

In addition, Kv3.1 mRNA is found in the substantia nigra (SN) (Weiser et al. 1995) and Kv3.2 channels are thought to be expressed on SN dopaminergic neurons (Dufour et al. 2014). AUT1, a K3.1/3.2 positive modulator from the same chemical series as AUT00206, modulated firing frequency and action potential properties of dopamine neurons in a ClockΔ19 mouse model of ventral tegmental area (VTA) driven mania (Parekh et al. 2018). This suggests that, in addition to effects on PV interneurons, AUT00206 could also act directly on cells within the SN and VTA to modulate the activity of dopamine projections to the striatum. AUT00206 may be acting through either, or a combination of both, of these two potential mechansims of action to underlie our findings.

## Conclusions

This study provides the first evidence that AUT00206, a Kv3.1/3.2 channel modulator, can modulate striatal reward circuitry in patients with schizophrenia. These results support further evaluation of AUT00206 as a novel, non-dopaminergic treatment for schizohrenia.

## Supplementary Information

Below is the link to the electronic supplementary material.

Supplementary file1 (PDF 305 KB)
