# Supplementary material for: The effects of AUT00206, a novel Kv3.1/3.2 potassium channel modulator, on task-based reward system activation: a test of mechanism in schizophrenia
Source: Psychopharmacology (Berl). 2022 Sep 12;239(10):3313–23. doi: 10.1007/s00213-022-06216-3 (PMC9481488; doi:10.1007/s00213-022-06216-3)
Supplement: Supplementary file 1 — Supplementary file1 (PDF 305 KB) [file 213_2022_6216_MOESM1_ESM.pdf]

Springer
the language of science

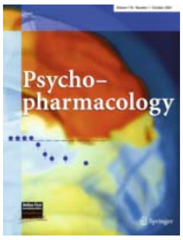


# *Conflict of Interest Disclosure Form*

It is the policy of the *Psychopharmacology* to ensure balance, independence, objectivity, and scientific rigor in the Journal. All authors are expected to disclose to the readers any real or apparent conflict(s) of interest that may have a direct bearing on the subject matter of the article. This pertains to relationships with pharmaceutical companies, biomedical device manufacturers or other corporations whose products or services may be related to the subject matter of the article or who have sponsored the study.

The intent of the policy is not to prevent authors with a potential conflict of interest from publication. It is merely intended that any potential conflict should be identified openly so that the readers may form their own judgements about the article with the full disclosure of the facts. It is for the readers to determine whether the authors' outside interest may reflect a possible bias in either the exposition or the conclusions presented.

The corresponding author will complete and submit this form, along with his or her submission, to the responsible Editor on behalf of all authors listed below.

*Article Title* **The effects of AUT00206 a novel Kv3.1/3.2 potassium channel modulator on task-based reward system activation: a test-of-mechanism in schizophrenia.**

*Authors* **Stephen J. Kaar, Ilinca Angelescu, Matthew M Nour, Tiago Reis Marques, Alice Sharman, Anil Sajjala, John Hutchison, Philip McGuire, Charles Large, Oliver D. Howes.**

Please note that a conflict of interest statement is published with each paper.

I certify that there is no actual or potential conflict of interest in relation to this article. If any conflict exists, please define hereafter:

*Conflict (if none, "None" or describe financial interest/arrangement with one or more organizations that could be perceived as a real or apparent conflict of interest in the context of the subject of this article):*

**This study was provided with funding from Innnovate UK. SK received travel expenses to attend a scientific meeting arranged by Autifony. IA has nothing to disclose. MN has nothing to disclose. TRM has received honoraria for speaking and chairing engagements from Lundbeck, Janssen, and Astellas. PM has nothing to disclose. OH is a part-time employee of H. Lundbeck A/S and has received investigator-initiated research funding from and/or participated in advisory/ speaker meetings organised by Angellini, Autifony, Biogen, Boehringer-Ingelheim, Eli Lilly, Heptares, Global Medical Education, Invicro, Jansenn, Lundbeck, Neurocrine, Otsuka, Sunovion, Rand, Recordati, Roche and Viatris/ Mylan. Dr Howes has a patent for the use of dopaminergic imaging. AS, AS, JH and CL are employees and shareholders in Autifony.**

Name **Stephen Kaar**

Signature ………………………………… Date **08/AUG/2022**

---

**Please scan the completed and signed form and then submit it as an electronic file along with your manuscript. No manuscripts will be accepted for review without this form.**

http://www.springer.com/journal/213

Psychopharmacology
Coordinating Editor: Robbins, T.W.; Baunez, C.; Janak, P.H. - Principal Editor: de Wit, H.; Curran, V.H.; Morrow, A.L.; Hashimoto, K.; Howes, O.; Floresco, S.B.; D'Souza, D.
ISSN: 0033-3158 (print version)
ISSN: 1432-2072 (electronic version)
Journal no. 213
